# An Unusual Cause of Cranial Dural Thickening

**DOI:** 10.1155/2020/8877738

**Published:** 2020-10-29

**Authors:** Jing Ming Yeo, Donald MacArthur, Jillian Davis, Ian Scott, Bruno Gran

**Affiliations:** ^1^Department of Neurology, Queen's Medical Centre, Nottingham University Hospitals NHS Trust, Derby Road, Lenton, Nottingham NG7 2UH, UK; ^2^Department of Neurosurgery, Nottingham University Hospitals NHS Trust, Nottingham NG7 2UH, UK; ^3^Department of Pathology, Nottingham University Hospitals NHS Trust, Nottingham NG7 2UH, UK; ^4^Department of Neurology, Nottingham University Hospitals NHS Trust, Nottingham NG7 2UH, UK

## Abstract

We describe an unusual cause of cranial dural thickening in an elderly female with a chronic meningeal inflammatory process. A 70-year-old ethnically Chinese, Singaporean female presented with a history of chronic daily headache with no other meningeal signs. Serial MRI brains showed progressive pachymeningeal and leptomeningeal enhancement in the left frontal region with underlying vasogenic oedema, similar appearances in the right frontal region to a lesser extent, and persistent inflammatory changes in her bilateral paranasal sinuses. Investigative work-up showed a chronically raised ESR with a normal CRP, negative ANCA, and a chronically raised serum IgA kappa paraprotein. Bone marrow trephine biopsy was suggestive of a low level plasma cell disorder. Olfactory cleft biopsy showed no evidence of IgG4-related disease or vasculitis and no significant plasma cell infiltrate. Histopathological examination from a meningeal biopsy revealed a diagnosis of an en-plaque meningioma (the WHO, 2016; Grade I) causing an unusual granulomatous reaction. We discuss the radiological and histological relations of this rare form of meningioma. Clinicians can consider en-plaque meningioma in the differential diagnosis of linear dural thickening and enhancement.

## 1. Introduction

Involvement of cranial dura mater often steers one to look for inflammatory causes such as granulomatosis with polyangiitis, neurosarcoidosis, rheumatoid, or IgG4-related disease; infective causes such as syphilis, mycobacterial or fungal pathology; or malignancy such as dural metastases or lymphoma [1]. Here, we describe a case of an elderly female with a slowly progressive cranial pachy- and leptomeningeal process revealed on meningeal biopsy to be an en-plaque meningioma causing an unusual granulomatous reaction.

## 2. Case Presentation

**Figure 1:**
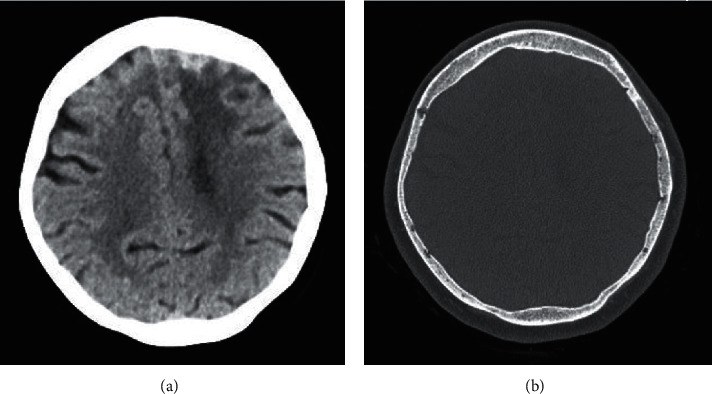
CT head: (a) left frontal extra-axial lesion with patchy calcification and vasogenic oedema; (b) CT bone window setting showing osteopenia but no bony erosion or hyperostosis.

**Figure 2:**
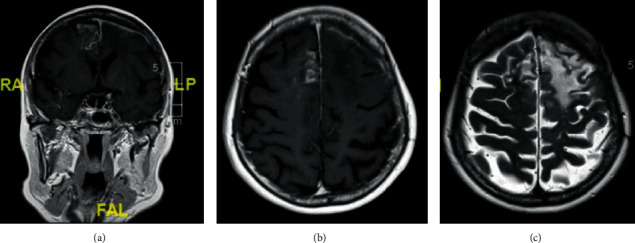
MRI brain. (a, b) Coronal and axial T1 postcontrast images showing pachymeningeal enhancement in the left frontal region and leptomeningeal enhancement in the left superior and middle frontal sulci, with vasogenic oedema. A nodular leptomeningeal enhancement is noted within the right parafalcine and superior frontal sulci with minimal underlying vasogenic oedema. There was no diffusion restriction. (c) Axial FLAIR image showing the extent of the vasogenic oedema.

A 70-year-old ethnically Chinese female presented via the Emergency Department with a 2-week history of shortness of breath, productive cough, and confusion. A chest radiograph showed focal consolidation, and she was treated with antibiotics for community-acquired pneumonia and delirium. By day seven, her confusion had resolved. As part of the investigative work-up of her confusion, she received a CT head which showed a left frontal extra-axial lesion with patchy calcification (Figure 1). An outpatient MRI brain a month later demonstrated pachymeningeal and leptomeningeal enhancement bifrontally, with more marked changes on the left with underlying vasogenic oedema, and inflammatory opacification of the paranasal sinuses (Figure 2).

She was subsequently admitted to the neurology ward for further assessment. She reported a one year exacerbation in the intensity of her chronic left frontal headache which she has had since her 30 s. They were described as a left frontal ache with migrainous features of nausea and photophobia, with no raised pressure features. She also described a reduced sense of smell and taste for 20 years which transiently improves on receiving prednisolone for exacerbation of asthma. There were no systemic features of arthralgia, weight loss, fevers, rash, or haemoptysis. Past medical history included childhood asthma, allergic contact dermatitis, and hypertension. Medication history included steroid and salbutamol inhalers, enalapril, and paracetamol. She was born in Singapore and has been a resident in the UK for 40 years. Neurological examination was normal with no nuchal rigidity, ophthalmic abnormalities, or cranial nerve signs.

Further investigations revealed a chronically raised ESR (35–63 mm/h) for the past eight years with a normal CRP. She also had a chronically raised serum total IgE (137 kU/L) with a normal eosinophil count. Serum ANA, ANCA, rheumatoid factor, double-stranded DNA, anti-Ro, La, Sm, RNP, Jo1, and Scl-70 were negative. She had a serum IgA kappa paraprotein band of 14 g/L with normal serum-free light-chain ratio, beta2-microglobulin, renal function, calcium, and albumin levels. Serum IgG subclasses including IgG4 were normal. Serum fungal and cerebrospinal fluid (CSF) tuberculosis cultures were negative. Two sets of CSF examination showed normal cell count, protein and glucose levels, negative PCR for herpes simplex, varicella zoster and enteroviruses, and no neoplastic cells. CT chest, abdomen, and pelvis were normal. CT sinus showed chronic rhinosinusitis, and an olfactory cleft biopsy showed inflammatory mucosal changes with no evidence of IgG4-related disease or vasculitis. Bone marrow biopsy showed 10% plasma cells on trephine biopsy and 7% plasma cells on aspirate, and borderline between monoclonal gammopathy of uncertain significance (MGUS) and myeloma. MRI whole body showed diffuse osteopenia but no active myelomatous lesions.

**Figure 3:**
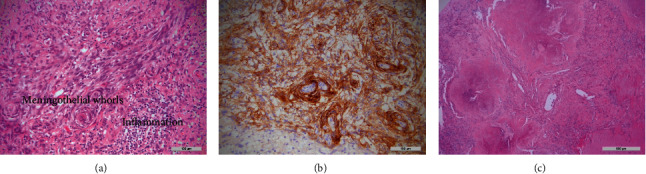
Histopathological examination from left frontal meningeal biopsy: (a) H&E stain demonstrating meningothelial whorls and associated chronic inflammation, (b) epithelial membrane antigen (EMA) stain confirming the meningothelial phenotype, and (c) H&E stain showing necrobiotic granulomata at the periphery of the lesion.

Serial MRI brains over two years showed a slow progression in the left frontal convexity pachymeningeal and leptomeningeal thickening and enhancement and bifrontal gliosis. She underwent an open left frontal meningeal biopsy two years into her initial presentation. Histopathological examination revealed a meningothelial neoplasm with a mitotic count of <4/10 high power fields and a low Ki67 labelling index (Figure 3). It also showed the presence of necrobiotic granulomata associated with elastotic degeneration. This was interpreted as an en-plaque meningioma (the WHO, 2016; Grade I) with associated granulomatous and fibroblastic reaction. Immunohistochemistry showed no tumoural expression of GFAP, CD34, STAT6, SMA, Desmin, MUC4, Cam5.2, or MNF116. Bacterial, fungal, and mycobacterial stains were negative.

It was discussed with the patient that she has a benign slow-growing growth within the meninges which occupies much of her left frontal lobe area and it would be difficult to safely remove the whole plaque. She remains under conservative management with serial radiological and clinical monitoring. If the meningeal areas were to grow more as a globular lump with associated symptoms, targeted resection or other forms of localised treatment may be considered.

## 3. Discussion

This is a case of an elderly female with a chronic meningeal inflammatory process. Her atopic tendency, paranasal sinus involvement, and chronically raised ESR initially raised the possibility of granulomatosis with polyangiitis; however, her serum ANCA was negative. The paranasal sinus inflammation was likely secondary to chronic rhinosinusitis unrelated to the meningeal process. Her IgA kappa paraprotein and bone marrow examination were suggestive of low level plasma cell disorder. However, meningeal involvement in plasma cell disorders often indicates advanced disease and this was inconsistent with the rest of her clinical presentation.

Meningiomas are classically morphologically globular with a rounded body growing inwards from the dura with a wide dural base and a dural tail. En-plaque meningioma constitutes 2–4% of all intracranial meningiomas and is characterised by its sheet-like growth along the meninges and invasion of the dura mater [2]. Due to the lack of an associated mass, they can present a diagnostic challenge. They are commonly found on the sphenoid wing, and only less than 1% grow on the frontal or temporal bones [3, 4]. On CT heads, a higher proportion of en-plaque meningiomas demonstrate hyperostosis compared to classical meningiomas (13 to 49% compared to 4.5%) [5]. Some are thought to be reactive, and others are thought to be due to true tumour growth within the bone inducing periosteal new bone formation [6]. They can also be associated with inward bulging of the skull vault and vasogenic oedema adjacent to the hyperostotic bone [7]. On MRIs, en-plaque meningiomas appear as linear dural thickening with homogenous contrast enhancement due to the presence of tumour cells and chronic inflammation [8].

Despite being locally invasive, en-plaque meningiomas are usually WHO Grade I [9]. Li et al. reported 2 out of 37 cases of sphenoid wing en-plaque meningiomas which turn out to be grade II [10]. Grade I meningiomas without clinical or functional limitations are usually managed conservatively. If there are atypical features, surgical resection of the involved areas including the bone, dura, muscle, intracranial, and intraorbital components is considered. Postoperative radiotherapy is used to reduce the risk of recurrence [11]. Goyal et al. reported that 79% of the 28 patients in his cohort who did not develop tumour recurrence were in the group who received radiotherapy [12]. Park et al. found that progression-free survival at 5 years was significantly higher in patients undergoing postoperative radiotherapy (58.7% versus 44.3%) [11]. Prognosis is generally favourable if excision is complete. However, total removal of sphenoid wing en-plaque meningioma is difficult due to extensive dural and bone involvement, and this corresponds to a high recurrence rate [13].

In cases of diagnostic uncertainty, a meningeal biopsy in consultation with the neurosurgery team can be pivotal in clinching the diagnosis. We suggest that clinicians consider en-plaque meningioma in the differential diagnosis of linear dural thickening and enhancement, particularly in the context of the described clinicoradiological features.

## Data Availability

The data used to support the findings of this study are available from the corresponding author upon request.
